# IoT Device Identification Using Directional Packet Length Sequences and 1D-CNN

**DOI:** 10.3390/s22218337

**Published:** 2022-10-30

**Authors:** Xiangyu Liu, Yi Han, Yanhui Du

**Affiliations:** 1 College of Information and Cyber Security, People’s Public Security University of China, Beijing 100038, China; 2 First Research Institute of the Ministry of Public Security of PRC, Beijing 100048, China

**Keywords:** Internet of Things, device identification, deep learning, fingerprinting

## Abstract

With the large-scale application of the Internet of Things (IoT), security issues have become increasingly prominent. Device identification is an effective way to secure IoT environment by quickly identifying the category or model of devices in the network. Currently, the passive fingerprinting method used for IoT device identification based on network traffic flow mostly focuses on protocol features in packet headers but does not consider the direction and length of packet sequences. This paper proposes a device identification method for the IoT based on directional packet length sequences in network flows and a deep convolutional neural network. Each value in a packet length sequence represents the size and transmission direction of the corresponding packet. This method constructs device fingerprints from packet length sequences and uses convolutional layers to extract deep features from the device fingerprints. Experimental results show that this method can effectively recognize device identity with accuracy, recall, precision, and f1-score over 99%. Compared with methods using traditional machine learning and feature extraction techniques, our feature representation is more intuitive, and the classification model is effective.

## 1. Introduction

In recent years, the number of Internet of Things (IoT) devices in use has continued to proliferate. It is estimated that the number of IoT devices will reach 75 billion by 2025 [1]. For both the traditional Internet and IoT, security remains an important issue. The challenge of IoT security comes from the heterogeneity of IoT devices [2,3], and the limited nature of their resources, such as processing ability, battery and bandwidth [4], for implementing traditional security solutions. Many security issues can be mitigated by identifying unknown devices in the local network, which enables appropriate security enforcement on a particular device. Besides cybersecurity application, IoT device identification is an important area of research that many other applications can further benefit from, especially the smart building domain. Researchers have developed many applications in this field recently, such as plug load automation and control [5], wireless communication [6], energy management [7], occupant-appliance interaction patterns, and abnormal traffic detection [8].

As IoT devices are constantly being installed in and removed from a network, it is essential to identify the device type or model for security concerns. IoT device identification (fingerprinting) is sometimes difficult due to the large variety of protocols used in devices. Commonly an IoT device should respond to queries about its identity, however, an unknown or compromised device might disguise itself as another device by sending false identity information. This behavior can be detected by fingerprinting techniques through passive network traffic analysis. Therefore, passive fingerprinting of IoT devices is of vital importance for securing IoT networks. In general, IoT device identification or fingerprinting is a multiclass classification problem. By training a classifier based on feature set extracted from network traffic traces, we can predict the type, model, and manufacturer of an unknown device when it first enters a network.

To find device features, many previous studies, such as IoT Sentinel [9], IoTSense [10], and IoTDevID [11], have used protocol-based and payload features; the details of their methods are covered in Section 2.1.

In this paper, we introduce a new method for IoT device identification (fingerprinting) that models feature extracted from the directions and lengths of packets in a network trace. Then, a classifier based on a convolutional neural network is used for device identification. The contributions of our work are as follows:

1. We propose a new feature extraction technique based on the directions and lengths of the packets in network traces which is fundamentally different from other IoT device identification methods. This may provide different insights compared to other feature extraction techniques used in this field.
2. Based on an evaluation of experimental results, our method performs better than previous work in terms of classification precision, recall, and F-1 score.

The rest of this paper is organized as follows: Section 2 reviews related works, and Section 3 defines our proposed method of data representation and classification. Section 4 reports the experimental results. These results are discussed in Section 5. Section 6 discusses limitations and concludes the paper.

## 2. Background and Related Work

This section reviews the previous work on IoT device identification and then presents some background on deep learning to provide a better understanding of our method.

### 2.1. IoT Device Identification

Research on IoT device identification, or fingerprinting, is still in the early stage due to the rapid development of the IoT industry. Many previous works have focused on device identification using fingerprinting methods to classify IoT devices. Device identification can be characterized as a classification problem in machine learning. The objective of the classification task may be either device type or model classification. First, features are extracted from raw network traffic data. Then the data in feature vector form are divided into training and testing sets, and different algorithms are used for training and testing. Many previous works differ in the feature extraction methods and machine learning algorithms used. Most of them have used features such as packet header and payload statistics, flow statistics, or timestamp features. Algorithms such as naïve Bayes, decision trees, random forest, k-nearest neighbors, and support vector machines are commonly used in these works. Table 1 summarizes various recent works on IoT device identification.

In [9], Miettinen et al. described a framework called IoT Sentinel for securing IoT devices using the fingerprinting approach. They extracted a set of 23 features from protocols in different layers of the network, IP options, packet content, and port class from 12 consecutive packets and finally obtained a feature set with a size of 23 × 12 = 276. Then, binary classifiers were trained for one device type versus the rest using the random forest algorithm. An experiment was performed over a set of 27 devices, and accuracies of 95% for 17 devices and 50% for 10 devices were achieved, resulting in an average of 85%.

In IoTSense [10], Bezawada et al. used part of the feature set from IoT Sentinel and another feature set from the payload. Specifically, IoTSense considers 17 protocol-based features and an additional three payload-related features. The fingerprints are produced by extracting 20 features from five packets, for a total of 100 features. This work achieved 99% average accuracy and 93–100% per-device recall using the k-NN, decision trees, and gradient boosting algorithms. This work on IoTSense considered a smaller number of devices than the work on IoT Sentinel (i.e., 10 vs. 31).

Sivanathan et al. [16] used a large dataset collected from 28 IoT devices over six months for IoT device classification. This work used eight features, namely flow volume, flow duration, average flow rate, device sleep time, server port numbers, DNS queries, NTP queries and cipher suites. Naïve Bayes and random forest were used together to construct the classifier. As a result, an accuracy of 99.88% was obtained. However, some of the features were too device specific, which could influence the classification results.

Meidan et al. [23] proposed a method to identify unauthorized IoT devices. Their dataset contained 17 devices. In total 334 features were extracted from each network traffic flow. A random forest was used for classification and as a result, unauthorized devices could be identified with an accuracy of 96%. The drawback of this study was that the features included application layer information, which is often encrypted in reality.

Shahid et al. [12] used bidirectional flow characteristics to identify different types of IoT devices. They used four different types of equipment: sensors, cameras, bulbs and sockets. From the algorithmic perspective, t-SNE used for dimension reduction, and a random forest was used for classification. This research finally achieved an accuracy of 99.9%.

Yin et al. [24] proposed IoT ETEI, an automated end-to-end IoT device identification method based on a CNN + BiLSTM deep learning model. Their method outperforms traditional methods with higher identification accuracy and less overhead. Even for IoT network traffic using encrypted protocols, the method can reach an identification accuracy of over 99%.

Similarly, to address the problem of device type and model identification, the author of this paper [15] used the ping operation to generate fingerprints of different IoT devices to distinguish real embedded devices from virtual or simulated embedded systems. For each ping, ping requests with an interval of 0.2 s were used to calculate the statistical characteristics based on time. Detection rates of 99.5% (using 25 pings) and 99.9% (using 200 pings) were obtained.

Oser et al. [14] used TCP timestamps to measure the clock offsets of different IoT device models for model identification. They used a total of 51 different models of 562 devices. When only the clock offset was used, the system could not identify most models. Therefore, the author used 12 other features obtained from timestamps in addition to the original clock offset, and finally obtained 97.03% accuracy, 94.64% recall rate and 99.76% accuracy rate.

Thangavelu et al. [13] proposed DEFT, a distributed device fingerprint identification system. In their method, an SDN network gateway is used to monitor and classify equipment locally. Statistical features based on packet headers and application layer protocols are extracted, and then a 15-min session is formed. To identify new equipment types, a clustering algorithm (k-means) and random forest are used. Perdisci et al. [25] analyzed the DNS application layer protocol to build fingerprints of IoT devices and used a method based on file retrieval to classify them.

Another related work in the context of IoT device model classification comes from Marchal et al. [19]. They presented Audi (Autonomous IoT Device-Type Identification), a system for identifying the types of IoT devices by passively analyzing periodic network communication. To identify periodic flows, a discrete Fourier transform was used to transform the time domain into the frequency domain. Then, 33 different features were calculated for each cycle. Their results were over 90% in F1-score and 98.2% in comprehensive accuracy. Msadek et al. [17] focused on encrypted traffic analysis, and they used a head sliding window to obtain statistical features. AdaBoost was used for classification, and an accuracy rate and F1-score of 95.5 were obtained.

Pinheiro et al. [20] proposed an IoT device and event identification technique based on packet length from encrypted traffic. Their solution utilized packet length statistics to identify IoT devices and events, including the mean packet length, the standard deviation, and the number of bytes transmitted over a one-second window. The method used only three statistics reducing the computational complexity for IoT traffic classification. The traffic classification algorithms used included k-NN, decision tree, random forest, support vector machine and majority voting. The results showed that the random forest algorithm could achieve up to 96% accuracy in the identification of devices on the UNSW dataset [18].

Duan et al. [22] proposed a practical IoT device identification system called ByteIoT based on the frequency distribution of bidirectional packet lengths. For ByteIoT, a k-NN classifier was applied. The authors evaluated ByteIoT on several datasets and the results showed that it achieved over 99% accuracy on the UNSW IEEE TMC 2018 dataset [16].

Following the same technical route, OConnor et al. [26] presented HomeSnitch, a communication classification framework designed for home IoT devices based on semantic behaviors (such as firmware upgrades, audio and video recording, and data uploading). HomeSnitch uses the adudump [27] traffic analysis tool to build an application layer model of the packet headers. Finally, 13 different features are extracted to describe the application layer data exchange. The authors used the YourThings [28] dataset for testing, and obtained 99.69% accuracy, 93.93% F1-score and 96.82% TPR. Trimananda et al. [29] performed similar research, and they proposed Ping-Pong, a tool for extracting signatures of packet events. This work addressed research on encrypted traffic and unknown protocols, and used a method based on clustering and statistical packet analysis. Hafeez et al. [30] proposed IoT-KEEPER, a system that uses an unsupervised learning method to identify device types and detect malicious behaviors. The methods used were fuzzy c-means and interpolation.

Using a more complex method based on deep learning, Ortiz et al. [31] proposed DeviceMien, a probability-based device identification framework that uses a stacked LSTM-autoencoder structure to automatically learn the characteristics of the original TCP packets, and then uses DBSCAN to cluster them. To test their model, the authors used two datasets, one public [16] and one private.

In commercial buildings, plug loads often account for up to one-third of the energy use. Some researchers have developed automatic smart plug load identification systems for enhancing the capabilities of existing load monitoring systems. Tekler et al. [21] proposed a near-real-time plug load identification approach that used low-frequency power data in office spaces. They applied a novel dynamic time window strategy during feature extraction. Then the proposed method was evaluated in online and offline settings for device identification using k-NN, random forest (RF), gradient boosting (GB), and bagging algorithms. As a result, the best online model achieved accuracies up to 93% using the bagging algorithm with a minimum dynamic time window of 5 min.

The above previous works have made important contributions to the IoT device identification, however most of the methods depend on certain packet header field values or related statistical values in the network traffic generated by IoT devices, which require manual feature engineering or predefined domain knowledge. Some related work also needs feature selection algorithms to select useful features from the feature pool. In this work, our method for IoT device identification uses the length and direction of continuous packets generated by a specific unknown device and then transforms it into sequence of packet length and direction, as the input of the deep learning model. The learning ability of the deep learning model is leveraged to extract features automatically. Extracting packet length and direction is simple and no other feature engineering techniques are needed. This method dramatically reduces the difficulty in manually extracting features, and has better generalization ability. In addition, the accuracy of our method is as good asor better than the best previous work.

### 2.2. Deep Learning

**Figure 1:**
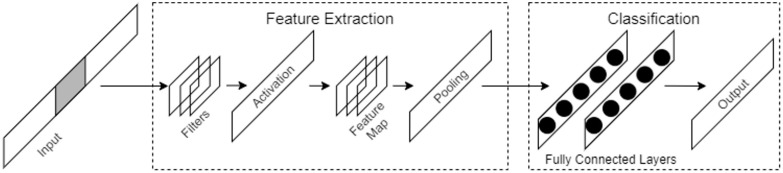
A basic CNN architecture.

Deep learning is a branch of machine learning methods; it refers to algorithms based on artificial neural network architectures that are used to perform representation learning on data [32,33]. The advantage of deep learning is the use of automated feature learning and hierarchical feature extraction algorithms in place of manual feature engineering. Convolutional neural networks (CNNs) consist of several convolutional layers and fully connected layers and include shared weights and pooling layers. This structure allows a CNN to use input data with a high-dimensional structure. CNNs produce better results in image and speech recognition compared with other neural network models. A CNN can also be trained using the backpropagation algorithm and with a smaller number of parameters than other deep feedforward neural networks. Figure 1 shows a block diagram of a traditional CNN. The diagram shows that there are two steps in a CNN: feature extraction and classification. Feature extraction is performed by convolutional layers and pooling layers and batch normalization and dropout are used to prevent overfitting. In classification, fully connected layers are used to classify the input as in a traditional multilayer perceptron (MLP).

## 3. Methodology

### 3.1. System Overview

**Figure 2:**
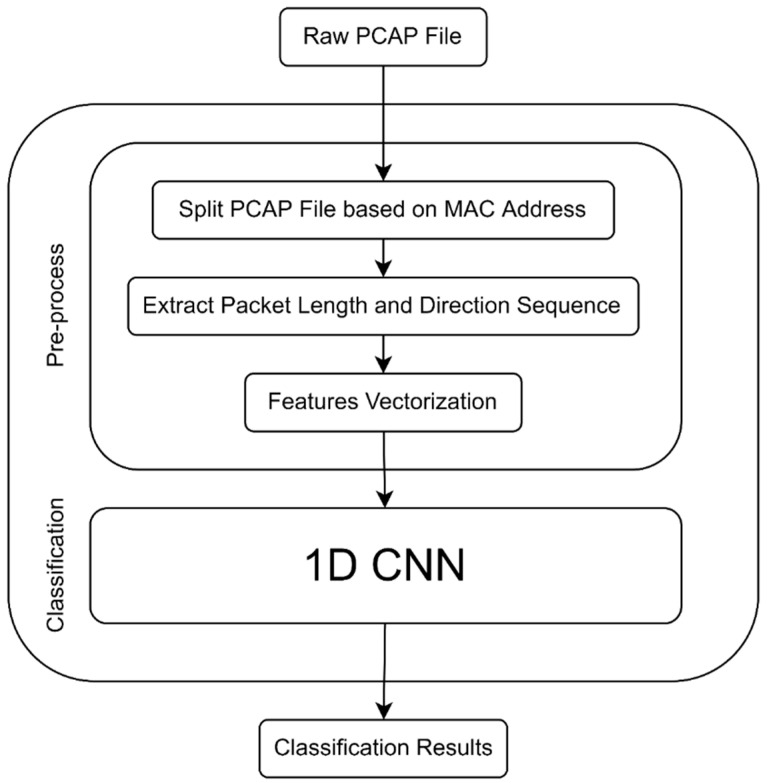
System diagram of the proposed method.

In our study, we propose a method for IoT device identification. Figure 2 presents our proposed system model. The first stage of the model shows when an unknown device joins the local IoT network. The model passively captures a sequence of network packets for the device. Then, a feature vector (fingerprint) is extracted from the network traces, and the unknown device can be identified using a classifier trained on a training set of known devices. Figure 2 shows the complete system workflow. We first describe the method used for preprocessing and representing the data of network traffic traces; the deep learning classifier used for final identification is introduced.

### 3.2. Data Preprocessing

IoT device identification can be seen as a supervised machine learning (or classification) problem. For a classification problem, first we should have feature vectors that can model the data of interest (network packet data); then, these feature vectors are fed into a classification model to obtain their predicted classes. In general, four categories of input features are used in network traffic classifiers: time-series, header, payload, and statistical features [34]. Each network packet includes a header and payload. For a layer-2 packet, if the payload is encrypted, the only information available to us is the metadata stored in the Ethernet header. Recent IoT device identification research has focused on extracting feature sets from packet headers and then using feature vectors obtained from individual packets for training and testing. Therefore, in the previous work, each packet was used as one data sample. In contrast, the proposed method utilizes several continuous packets and one such packet sequence is used as one data sample for device identification. This feature extraction and representation method is introduced below.

In this work, we use two time-series features, namely, packet length and direction, of N continuous packets. The feature vector is of length N with 1 channel in which packet length and direction are combined. As in [35], we define an outgoing packet from a device as having a positive value, whereas the incoming packet has a negative value. The original dataset is in PCAP format, and the network traffic is captured by software located in the gateway of a local network. The first step is aggregating packets generated by each device in chronological order according to the MAC address of each device. As a result, the PCAP file is split into different small files, and each small file contains all the packets generated by each device in a specific time period. For a small PCAP file, we extract the packet length and then combine the packet length and direction to obtain a numeric value for each packet. For example, 1400 denotes that this is an incoming packet of length 1400. Then a long sequence consisting of these numeric values is constructed in time order. To make a feature vector representing the device, the long sequence is sliced for every N packets to obtain multiple feature vectors of length N. The trailing packets are dropped for convenience. If the length of the feature vector is too long, this leads to more parameters in the classification model, and more computational resources are needed. In addition, it is also not good if the length is too short, as a lack of sufficient information will cause the method to fail. The optimal length N must be determined through experiments.

### 3.3. Convolutional Neural Networks

Convolutional neural networks have been widely used in image recognition. Data in image recognition task is high-dimensional tensor. Since the network traffic data are represented by one-dimensional sequence, inspired from sequence classification tasks such as DNA sequence classification [36] and heart sound signal processing [37], we adopt 1-D convolutional layers in the network design, in contrast to traditional image recognition applications. Another difference is that in traditional image classification, activation functions such as the sigmoid and rectified linear unit (ReLU) are widely used, but they do not work on negative values. Thus, the packet direction information would be lost if we were to use these functions; instead, activation functions such as hyperbolic tangent (tanh), leaky ReLU, and exponential linear unit (ELU) can deal with negative values. Among these three activation functions, we performed comparisons during hyperparameter tuning and found that ELU performed best.

**Figure 3:**
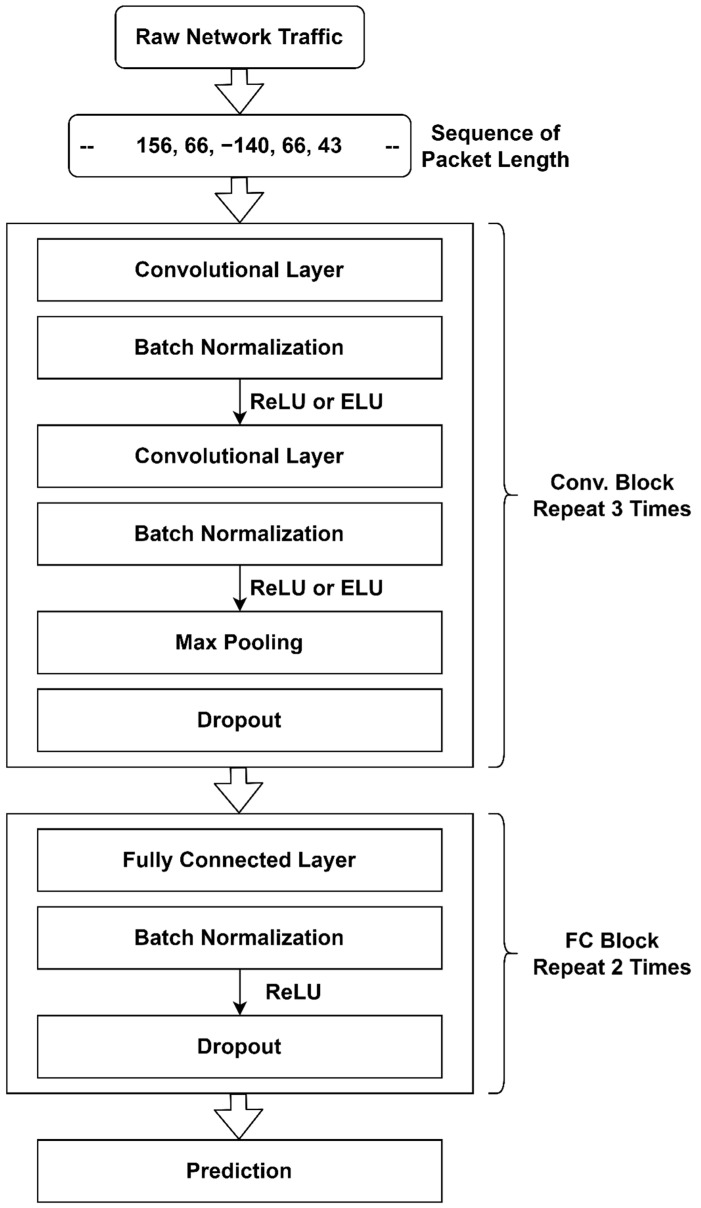
Block diagram of the proposed CNN model.

Our CNN model includes three convolutional blocks and two fully connected blocks. The three convolutional blocks look similar except for the number of filters and the kernel size. Each convolutional block comprises one convolutional layer, followed by batch normalization, then an activation function (ReLU or ELU), followed by another convolutional layer, batch normalization, and an activation function Finally, max pooling and dropout are used. This block is repeated three times with a different kernel size each time. In each fully connected (FC) block, a fully connected layer is followed by batch normalization, ReLU activation, and dropout. The FC block structure is repeated twice with different numbers of neurons in the fully connected layers. The block diagram of our CNN model is shown in Figure 3.

### 3.4. CNN Hyperparameter Tuning

In a supervised classification task, many hyperparameters need to be tuned. such as the value of k in k-NN, or the number of hidden layers in MLP. Properly tuning the hyperparameters allows model not only to fit the training data but also to generalize on the test data that it has not been trained on. We performed an extensive search in the hyperparameter space to find the better hyperparameters for our model. The model was built block by block in each layer. For each layer of the CNN model, we performed an experiment by varying the hyperparameters-and then chose the hyperparameters that gave the best performance. The search spaces and final values after hyperparameter tuning are shown in Table 2.

Table 3 lists the input/output shape, kernel shape, and number of parameters for each layer of this CNN model.

## 4. Experimental Evaluation

In this section, we describe the experimental setup and the dataset on which the identification tests were carried out. The results were obtained in two different scenarios: (1) classifying IoT devices into 7 categories and (2) classifying 18 IoT device models. The results for these two scenarios are shown below.

### 4.1. Experimental Setup and Datasets

Three different real device datasets available for public use that can be used for IoT device identification. Their names, creation year, and number of devices are as follows: Aalto University [9], 2016, 27 devices; UNSW dataset [18], 2016, 28 devices; IoTFinder [25], 2019, 51 devices. During the selection of the dataset to be used in our experiments, we found that the Aalto University dataset contains only network traffic from the device installation process. Although this installation process was repeated 20 times to increase the quantity of data, this dataset is still too small compared with the others. The UNSW dataset was built by collecting network traffic data of IoT devices in normal working environments rather than during the device installation process. The raw PCAP file size is 11.3 GB, which is large enough for deep learning evaluation. The IoTFinder dataset relies only on DNS traffic to identify IoT devices, which is not suitable for our objective. Thus, considering the advantages of the UNSW dataset, we chose to use this dataset for all the evaluations and analyses presented below. This dataset contains various types of devices, including lights, cameras, hubs, and healthcare devices. Table 4 provides detailed information about the devices. The TP-Link router is a gateway to the Internet. The WAN interface of the router was connected to the Internet, and the IoT devices were connected to the LAN or WLAN interfaces. Some software was installed on the gateway such as the tcpdump tool for collecting the network traffic of all devices. Then the collected network traffic was stored in PCAP files. We parsed the PCAP files and extracted informative features in accordance with the MAC address of each device.

The dataset is organized by date, with one PCAP file for one day. In total, we downloaded 20 PCAP files corresponding to 31 device types. However, the original dataset contains several devices such as iPhones, laptops, and routers which cannot be categorized as IoT devices. Because the objective of this research is to study the relationship between network traffic behavior and IoT device type or model, we ignored these non-IoT devices for the purity of the dataset. We used the MAC addresses to aggregate the data from the raw PCAP files. In addition, the MAC addresses of several devices could not be found in the PCAP files and thus were also ignored. After cleaning the data, we finally obtained 18 devices. The PCAP file for each day was parsed using the MAC addresses of the devices. Finally, a long sequence of packet direction and size was generated for each device. We cut each long sequence into slices every 500 entries to obtain our feature vectors, as described in Section 3.1. The statistics on the distribution of the data samples for each device after the completion of the above data preprocessing steps are shown in Table 5.

Three metrics were used for the performance evaluation of our model. The definitions of metrics are listed below; they are precision, recall, and F1-score. TP is true positive, FP is false negative, FN is false negative.

$$precision=\frac{TP}{TP+FP}$$

$$recall=\frac{TP}{TP+FN}$$

$$F_{1}\text{-}score\;=\;\frac{2\;\cdot \;precision\;\cdot \;recall}{precision\;+\;recall}$$

### 4.2. Device Category Identification

In this experiment, we explored the capability of our model to classify devices into different categories. For this experiment, we generated a new dataset from the original dataset by grouping the devices into different device categories, e.g., hubs or cameras. The statistics of this dataset are shown in Table 6. This dataset consists of the categories from Table 4.

**Figure 4:**
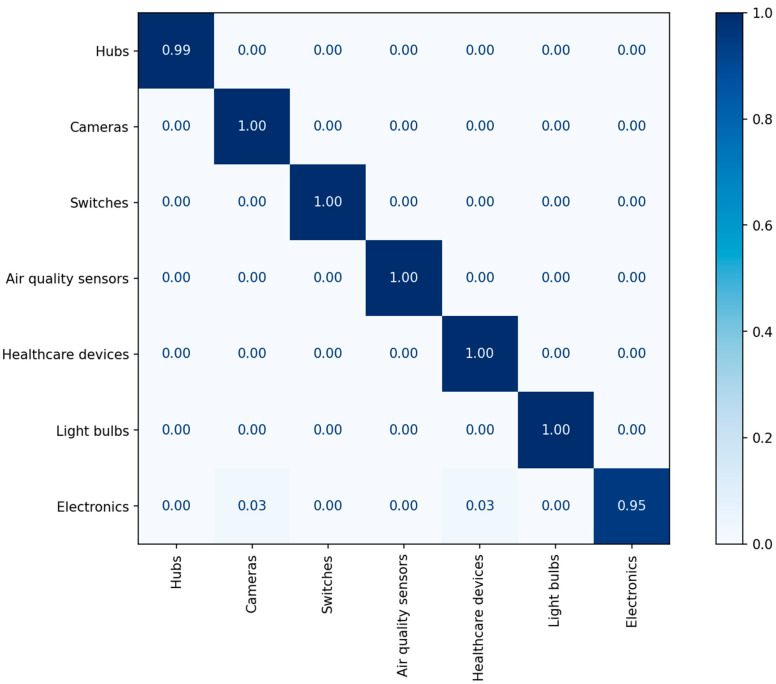
The confusion matrix of the classification results for category identification.

We used the CNN model described in Table 1. The generated dataset was split at a ratio of 80%:10%:10% for training, validation, and testing. The experimental results including precision, recall, and F1-score are listed in Table 7, and Figure 4 illustrates the confusion matrix of the classification results.

### 4.3. Device Model Identification

**Figure 5:**
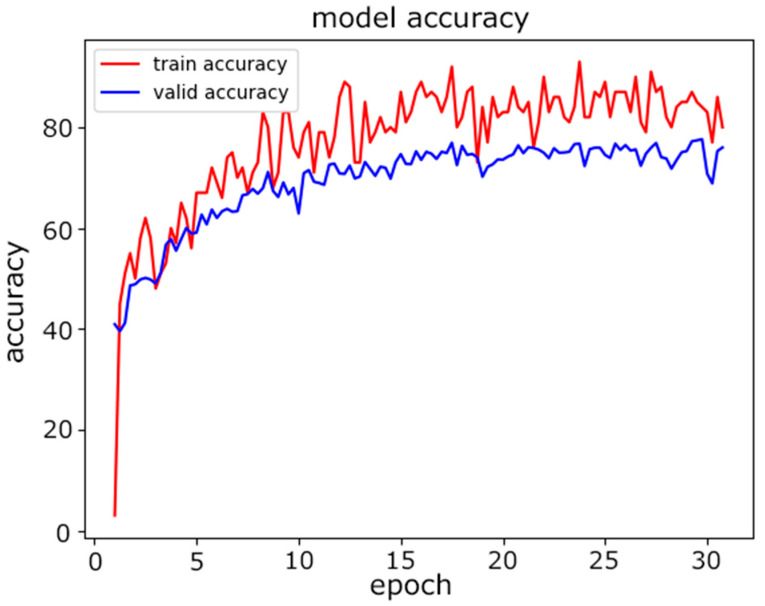
MLP model accuracy.

**Figure 6:**
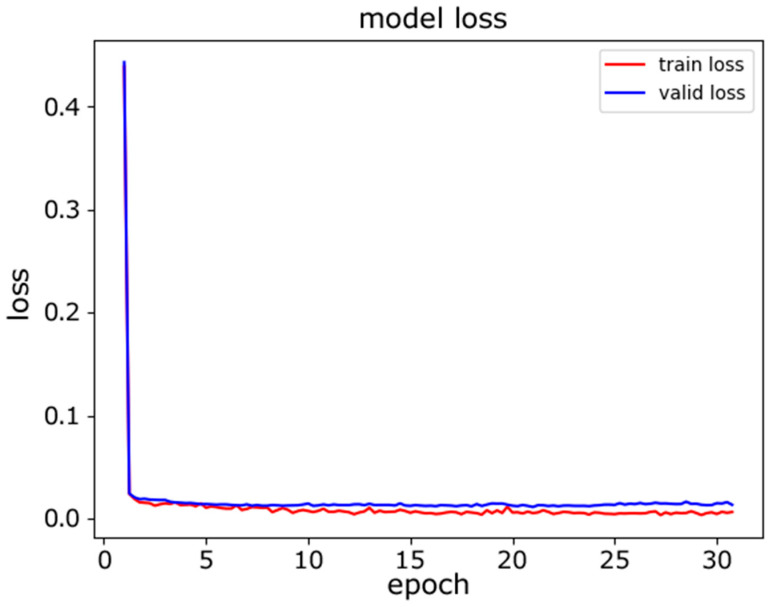
MLP model loss.

In this experiment, we evaluated the classification accuracy of the proposed model for device model fingerprinting. The goal of the classifier was to distinguish distinct devices. The experiment was conducted with two models for comparative study. First, we used a traditional neural network model, an MLP, for classification. The architecture of the MLP used is described in Table 8. The dataset was split at a ratio of 80%:10%:10% for the training, validation, and testing. Figure 5 and Figure 6 show the training progress of the MLP from the perspectives of accuracy and loss, and it can be seen that the accuracy curve was not stable in the last several epochs of training.

**Figure 7:**
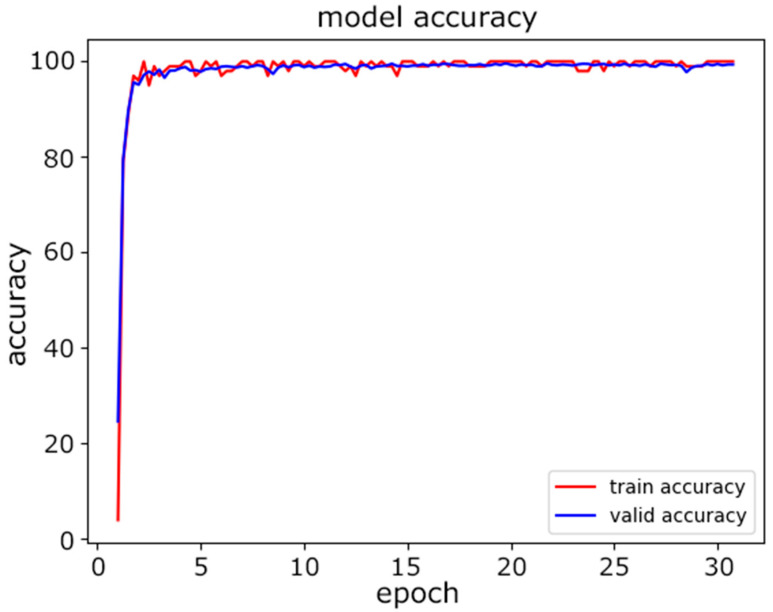
CNN model accuracy.

**Figure 8:**
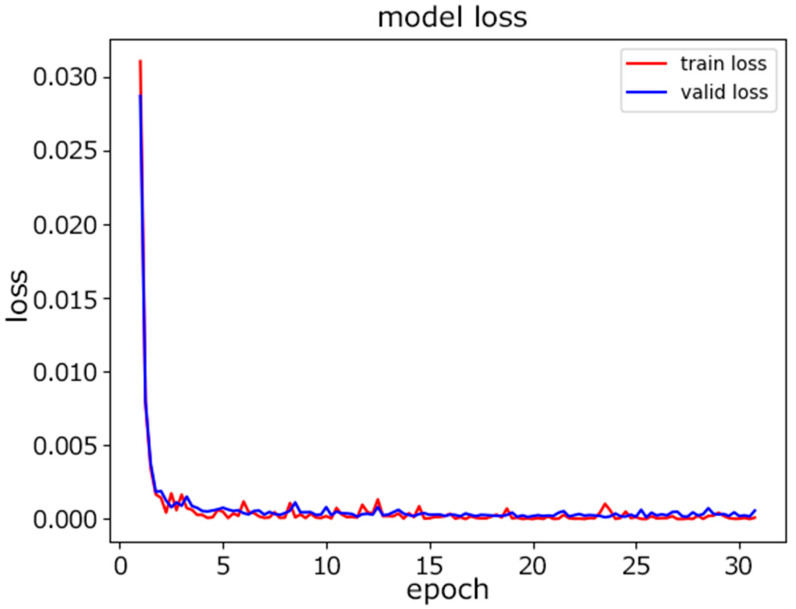
CNN model loss.

Second, the proposed CNN model was tested on the same data. The dataset was again split at a ratio of 80%:10%:10% for the training, validation, and testing. Figure 7 shows the progress of training in terms of accuracy as the number of epochs increased. We selected the maximum number of epochs to be 30 to achieve the desired accuracy. Figure 8 illustrates how the loss was is reduced as the number of epochs increased. The loss we used during training was the cross-entropy loss. As the value of the loss decreased, the predictions improved.

**Figure 9:**
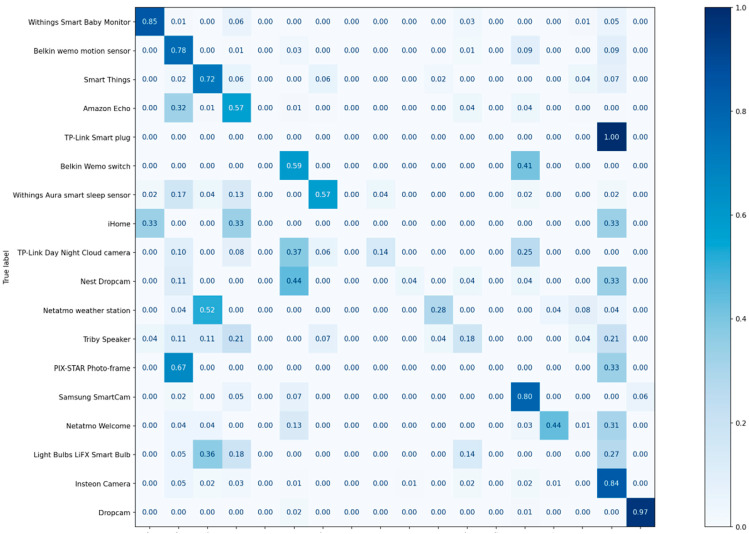
The confusion matrix of the classification results for the MLP.

**Figure 10:**
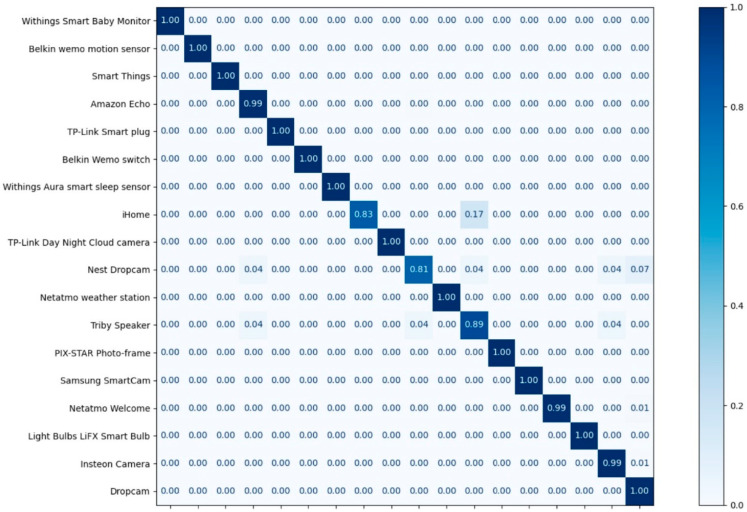
The confusion matrix of the classification results for the CNN.

Table 9 and Table 10 show the experimental results in terms of the precision, recall, and F1-score of each class of MLP and CNN, respectively. On average, the precision achieved is 99%, and the recall achieved is 99%. The F1-score is a measure that combines precision and recall; on average, the F1-score achieved was 99%. Figure 9 and Figure 10 illustrate the confusion matrices of the classification results.

## 5. Discussion and Limitation

In our study, we performed device identification by fingerprinting the packet length of network traffic flows via a deep learning algorithm. A certain number of successive packets from a specific device were used to construct a sequence that we took as a fingerprint. The experimental results show that this method is effective and efficient. For comparison, IoT Sentinel uses the first 12 packets during the device installation process to extract the feature vector, which is an approach that cannot be directly applied to the UNSW dataset. Thus, we cannot present a comparison with the results of IoT Sentinel. The authors of IoTSense did not provide their private dataset, so we cannot reproduce their methods with the UNSW dataset. IoTDevID leverages feature extraction techniques similar to those of IoT Sentinel and IoTSense, with some modifications and performance enhancement. Therefore, the results of IoTDevID on the UNSW dataset are a good benchmark for comparison. Compared with previous work on the same dataset, namely, UNSW [18], the work of Msadek et al. [17], and IoTDevID [11], the proposed CNN model achieves superior performance in classification accuracy, meaning that this algorithm can identify IoT devices with very high accuracy. Compared with a shallow neural network (MLP), the accuracy of classification is also boosted significantly by leveraging deep learning (CNN). A performance comparison of those different methods is shown in Table 11.

The dataset we used in this study is unbalanced in terms of the number of data instances for each device. As a result, the classification precision for devices such as the iHome and TP-Link Smart plug is poor because of insufficient training data. One solution may be to use data augmentation techniques, but this is beyond the scope of this paper. Another limitation of deep learning is that the quantity of data needed to train a network increase as the network becomes deeper. Simply, the more complicated a model is, the more training data are needed. Therefore, deep learning models do not perform as well on small datasets. Traditional machine learning algorithms can usually perform well with fewer data.

Another limitation of this work is that to construct the feature vector, it is necessary to use 500 consecutive packets in a flow, making the latency of the identification system larger than that in previous works, such as IoT Sentinel and IoTSense which use only tens of packets. In the future, we will explore how to use a smaller feature vector size while maintaining high identification accuracy.

## 6. Conclusions

This work proposes an IoT device fingerprinting method that uses only the directions and lengths of packets in a sequence as input features. This method reduces the effort for manual feature engineering from packet metadata compared to many previous works. Moreover, it leverages deep learning techniques (specifically, a CNN) for more accurate IoT device identification. The proposed method can effectively recognize device identity with an accuracy of over 99%. The use of the CNN demands many more computational resources than previous works, but this issue can be solved by deploying the proposed fingerprinting system on a local network server or gateway rather than on IoT devices. In conclusion, we developed a fingerprinting method using only the directions and lengths of packets to summarize the network traffic of IoT devices. Our study shows that packet length and direction are important features of the network traffic generated by IoT devices and that IoT device identification tasks can be successfully performed with only these features. In addition, a CNN with one-dimensional convolutional layers is a powerful tool for processing sequence data of this kind. Our study proposes a new direction for fingerprinting IoT devices based on automatic feature extraction from raw data using deep learning rather than manual feature engineering.

There are possibly several future research directions for device identification. First, different kinds of representations for network traffic can be explored. For example, network traffic is represented by sequence in this work, it can also be transformed to image which contains more information. Second, beyond the device type and model identification, device behavior identification needs to be studied in fine granularity. Last but not least, fast online device identification systems need more exploit in the future.

## Figures and Tables

**Table 1:** Device identification solutions based on device fingerprinting.

Work	Year	Algorithms	Features	Dataset	Classes	Results
**[9]**	2017	RF	Packet header-based	[9]	Device Type	81.5% for 27 devices, 95% for 17 of them.
**[10]**	2018	Gradient boosting, kNN, DT	Header payload statistics	Private	Device Type	99% overall acc. 86–99% TPR
**[12]**	2018	t-SNE, RF	Flow statistics	Private	Device Type	99.9% acc. for 4 types
**[13]**	2018	k-means, RF	IoT protocol flow statistics	Private	Device Type	97% acc. 97% F1-score
**[14]**	2018	RF, SVM, MLP	Clock skew and timestamp features	Private	Device Model	97.03% precision, 94.6% recall, 99.76% acc. for 51 devices
**[15]**	2018	RF	Ping timestamp	Private	Real/Virtual Device	99.5% accuracy using 25 pings and 99.9% over 200 pings.
**[16]**	2019	RF	Flow/Packet statistics	[16]	Device Model	99.88% acc., 5.06% RRSE
**[17]**	2019	AdaBoost	Encrypted flow statistics	[18]	Device Model	95.5% acc. and F1-score
**[19]**	2019	Clustering + k-NN	Flow periods (DFT)	Private	Device Type	F1-score above 90% for 21/23% labels and 98.2% overall acc.
**[20]**	2019	RF	Packet length statistics	[18]	Device Model	96% overall acc.
**[21]**	2020	k-NN, RF, GB, Bagging	low frequency data	[21]	Device Type	accuracies up to 93% in online setting
**[22]**	2021	k-NN	Frequency distribution of packet lengths	[16]	Device Model	Over 99% acc.
**[11]**	2021	RF	Header payload statistics	[9,18]	Device Model	94–99% acc. using different aggregated packets

**Table 2:** Hyperparameters search space and final values for the CNN models.

**Hyperparameters**	**Search Space**	**Final**
**Input Dimension**	[100, 1000]	500
**Optimizer**	Adam, Adamax	Adam
**Learning Rate**	[0.0009, 0.01]	0.001
**Training Epochs**	[10, 50]	30
**Batch Size**	[16, 256]	100
**[Filter, Pool, Stride] Sizes**	[2, 16]	[8, 8, 4]
**Activation Function**	Tanh, ReLU, ELU	ELU
**Number of Filters**		
**Block 1 [Conv1, Conv2]**	[8, 64]	[32, 32]
**Block 2 [Conv3, Conv4]**	[32, 128]	[64, 64]
**Block 3 [Conv5, Conv6]**	[64, 256]	[128, 128]
**Pooling Layers**	[Min, Average, Max]	Max
**Number of FC Layers**	[1, 4]	2
**Hidden Units of FC Layers**	[128, 1024]	512
**Dropout [Pooling, FC1, FC2]**	[0.1, 0.8]	[0.1, 0.7, 0.5]

**Table 3:** Input/output/kernel shapes, and number of parameters for each layer of the model.

Block	Layer	Input Shape	Output Shape	Kernel Shape	Param #
**Conv. Block 1**	Conv1d	[-, 1, 500]	[-, 32, 493]	[8]	288
	BatchNorm1d	[-, 32, 493]	[-, 32, 493]	--	64
	ELU	[-, 32, 493]	[-, 32, 493]	--	--
	Conv1d	[-, 32, 493]	[-, 32, 486]	[8]	8224
	BatchNorm1d	[-, 32, 486]	[-, 32, 486]	--	--
	ELU	[-, 32, 486]	[-, 32, 486]	--	--
	MaxPool1d	[-, 32, 486]	[-, 32, 120]	8	--
	Dropout	[-, 32, 120]	[-, 32, 120]	--	--
**Conv. Block 2**	Conv1d	[-, 32, 120]	[-, 64, 113]	[8]	16,448
	BatchNorm1d	[-, 64, 113]	[-, 64, 113]	--	128
	ELU	[-, 64, 113]	[-, 64, 113]	--	--
	Conv1d	[-, 64, 113]	[-, 64, 106]	[8]	32,832
	BatchNorm1d	[-, 64, 106]	[-, 64, 106]	--	128
	ELU	[-, 64, 106]	[-, 64, 106]	--	--
	MaxPool1d	[-, 64, 106]	[-, 64, 25]	8	--
	Dropout	[-, 64, 25]	[-, 64, 25]	--	--
**Conv. Block 3**	Conv1d	[-, 64, 25]	[-, 128, 18]	[8]	65,664
	BatchNorm1d	[-, 128, 18]	[-, 128, 18]	--	256
	ELU	[-, 128, 18]	[-, 128, 18]	--	--
	Conv1d	[-, 128, 18]	[-, 128, 11]	[8]	131,200
	BatchNorm1d	[-, 128, 11]	[-, 128, 11]	--	256
	ELU	[-, 128, 11]	[-, 128, 11]	--	--
	MaxPool1d	[-, 128, 11]	[-, 128, 1]	8	--
	Dropout	[-, 128, 1]	[-, 128, 1]	--	--
**FC Block 1**	Flatten	[-, 128, 1]	[-, 128]	--	--
	Linear	[-, 128]	[-, 512]	--	66,048
	BatchNorm1d	[-, 512]	[-, 512]	--	1024
	ReLU	[-, 512]	[-, 512]	--	--
	Dropout	[-, 512]	[-, 512]	--	--
**FC Block 2**	Linear	[-, 512]	[-, 512]	--	262,656
	BatchNorm1d	[-, 512]	[-, 512]	--	1024
	ReLU	[-, 512]	[-, 512]	--	--
	Dropout	[-, 512]	[-, 512]	--	--
**Output**	Linear	[-, 512]	[-, 18]	--	9234
**Total parameters: 595,538**					

**Table 4:** List of IoT devices in the dataset.

Category	Device	Wireless/Wired
**Hubs**	Smart Things	Wired
	Amazon Echo	Wireless
**Cameras**	Netatmo Welcome	Wireless
	TP-Link Day Night Cloud camera	Wireless
	Samsung SmartCam	Wireless
	Dropcam	Wireless
	Insteon Camera	Wired/Wireless
	Nest Dropcam	Wireless
	Withings Smart Baby Monitor	Wired
**Switches & Triggers**	Belkin Wemo switch	Wireless
	TP-Link Smart plug	Wireless
	iHome	Wireless
	Belkin wemo motion sensor	Wireless
**Air quality sensors**	Netatmo weather station	Wireless
**Healthcare devices**	Withings Aura smart sleep sensor	Wireless
**Light Bulbs**	LiFX Smart Bulb	Wireless
**Electronics**	Triby Speaker	Wireless
	Pix-Star Photo-frame	Wireless

**Table 5:** Statistics about the number of data instances for each category.

Device	Data Instances
**Dropcam**	8266
**Amazon Echo**	2009
**Samsung SmartCam**	1915
**Insteon Camera**	1419
**Belkin wemo motion sensor**	1268
**Withings Smart Baby Monitor**	1116
**Belkin Wemo switch**	935
**Smart Things**	931
**Netatmo Welcome**	874
**Withings Aura smart sleep sensor**	647
**TP-Link Day Night Cloud camera**	526
**Nest Dropcam**	299
**Netatmo weather station**	272
**LiFX Smart Bulb**	251
**Triby Speaker**	248
**PIX-STAR Photo-frame**	93
**iHome**	80
**TP-Link Smart plug**	45

**Table 6:** Statistics on the number of data instances for each category.

Device	Data Instances
**Hubs**	2940
**Cameras**	14,415
**Switches & Triggers**	2328
**Air quality sensors**	272
**Healthcare devices**	647
**Light bulbs**	251
**Electronics**	341

**Table 7:** The precision, recall, and F1-score results and numbers of test data for category.

Category	Precision	Recall	F1-Score	Support
**Hubs**	1.00	0.99	1.00	292
**Cameras**	1.00	1.00	1.00	1439
**Switches**	1.00	1.00	1.00	248
**Air quality sensors**	1.00	1.00	1.00	25
**Healthcare devices**	0.98	1.00	0.99	54
**Light bulbs**	1.00	1.00	1.00	22
**Electronics**	0.97	0.95	0.96	40
**Weighted average**	1.00	1.00	1.00	2120

**Table 8:** MLP layer characteristics.

Layer	Neurons	Input Dimension	Activation
**1**	512	500	ReLU
**2**	128	-	ReLU
**3**	18	-	sigmoid

**Table 9:** The precision, recall, and F1-score results and the numbers of test data for the MLP.

Device	Precision	Recall	F1-Score	Support
**Withings Smart Baby Monitor**	0.96	0.85	0.90	104
**Belkin wemo motion sensor**	0.48	0.78	0.60	138
**Smart Things**	0.64	0.72	0.68	83
**Amazon Echo**	0.71	0.57	0.63	209
**TP-Link Smart plug**	0.00	0.00	0.00	4
**Belkin Wemo switch**	0.43	0.59	0.50	100
**Withings Aura smart sleep sensor**	0.74	0.57	0.65	54
**iHome**	0.00	0.00	0.00	6
**TP-Link Day Night Cloud camera**	0.78	0.14	0.23	51
**Nest Dropcam**	0.50	0.04	0.07	27
**Netatmo weather station**	0.70	0.28	0.40	25
**Triby Speaker**	0.20	0.18	0.19	28
**PIX-STAR Photo-frame**	0.00	0.00	0.00	12
**Samsung SmartCam**	0.61	0.80	0.70	179
**Netatmo Welcome**	0.91	0.44	0.59	94
**Light Bulbs LiFX Smart Bulb**	0.00	0.00	0.00	22
**Insteon Camera**	0.56	0.84	0.67	130
**Dropcam**	0.99	0.97	0.98	854
**weighted average**	0.77	0.76	0.75	2120

**Table 10:** The precision, recall, and f1-score results and the numbers of test data for the CNN.

Device	Precision	Recall	F1-Score	Support
**Withings Smart Baby Monitor**	1.00	1.00	1.00	104
**Belkin wemo motion sensor**	0.99	1.00	1.00	138
**Smart Things**	1.00	1.00	1.00	83
**Amazon Echo**	0.99	0.99	0.99	209
**TP-Link Smart plug**	1.00	1.00	1.00	4
**Belkin Wemo switch**	1.00	1.00	1.00	100
**Withings Aura smart sleep sensor**	1.00	1.00	1.00	54
**iHome**	1.00	0.83	0.91	6
**TP-Link Day Night Cloud camera**	1.00	1.00	1.00	51
**Nest Dropcam**	0.81	0.81	0.81	27
**Netatmo weather station**	1.00	1.00	1.00	25
**Triby Speaker**	0.93	0.89	0.91	28
**PIX-STAR Photo-frame**	1.00	1.00	1.00	12
**Samsung SmartCam**	1.00	1.00	1.00	179
**Netatmo Welcome**	1.00	0.99	0.99	94
**Light Bulbs LiFX Smart Bulb**	1.00	1.00	1.00	22
**Insteon Camera**	0.98	0.99	0.99	130
**Dropcam**	1.00	1.00	1.00	854
**weighted average**	0.99	0.99	0.99	2120

**Table 11:** Comparison of different methods.

Work	Method	Accuracy
**IoTDevID [11]**	Random Forest	98.8%
**UNSW [18]**	Random Forest	95%
**Msadek et al. [17]**	AdaBoost	95.5%
**Pinheiro et al. [20]**	Random Forest	96%
**This work**	MLP	77%
**This work**	CNN	99%

## Data Availability

Publicly available datasets were analyzed in this study. This data can be found here: https://iotanalytics.unsw.edu.au/iottraces (accessed on 10 October 2022).

## References

[1] Internet of Things (IoT) Connected Devices Installed Base Worldwide from 2015 to 2025. https://www.statista.com/statistics/471264/iot-number-of-connected-devices-worldwide/.

[2] Chaabouni N., Mosbah M., Zemmari A., Sauvignac C., Faruki P. (2019). Network Intrusion Detection for IoT Security Based on Learning Techniques. IEEE Commun. Surv. Tutor..

[3] Hussain F., Hussain R., Hassan S.A., Hossain E. (2020). Machine Learning in IoT Security: Current Solutions and Future Challenges. IEEE Commun. Surv. Tutor..

[4] Zarpelão B.B., Miani R.S., Kawakani C.T., de Alvarenga S.C. (2017). A survey of intrusion detection in Internet of Things. J. Netw. Comput. Appl..

[5] Tekler Z.D., Low R., Yuen C., Blessing L. (2022). Plug-Mate: An IoT-based occupancy-driven plug load management system in smart buildings. Build. Environ..

[6] Wang D., Wang H., Fu Y. (2021). Blockchain-based IoT device identification and management in 5G smart grid. EURASIP J. Wirel. Commun. Netw..

[7] Hamdi M., Messaoud H., Bouguila N. (2020). A new approach of electrical appliance identification in residential buildings. Electr. Power Syst. Res..

[8] Salman O., Elhajj I.H., Chehab A., Kayssi A. (2022). A machine learning based framework for IoT device identification and abnormal traffic detection. Trans. Emerg. Telecommun. Technol..

[9] Miettinen M., Marchal S., Hafeez I., Asokan N., Sadeghi A.-R., Tarkoma S. IoT SENTINEL: Automated Device-Type Identification for Security Enforcement in IoT. Proceedings of the 2017 IEEE 37th International Conference on Distributed Computing Systems (ICDCS).

[10] Bezawada B., Bachani M., Peterson J., Shirazi H., Ray I., Ray I. (2018). Iotsense: Behavioral fingerprinting of iot devices. arXiv.

[11] Kostas K., Just M., Lones M.A. (2021). IoTDevID: A Behaviour-Based Fingerprinting Method for Device Identification in the IoT. arXiv.

[12] Shahid M.R., Blanc G., Zhang Z., Debar H. IoT devices recognition through network traffic analysis. Proceedings of the 2018 IEEE International Conference on Big Data (Big Data).

[13] Thangavelu V., Divakaran D.M., Sairam R., Bhunia S.S., Gurusamy M. (2018). DEFT: A distributed IoT fingerprinting technique. IEEE Internet Things J..

[14] Oser P., Kargl F., Lüders S. Identifying devices of the internet of things using machine learning on clock characteristics. Proceedings of the International Conference on Security, Privacy and Anonymity in Computation, Communication and Storage.

[15] Selis V., Marshall A. (2018). A classification-based algorithm to detect forged embedded machines in IoT environments. IEEE Syst. J..

[16] Sivanathan A., Gharakheili H.H., Loi F., Radford A., Wijenayake C., Vishwanath A., Sivaraman V. (2018). Classifying IoT devices in smart environments using network traffic characteristics. IEEE Trans. Mob. Comput..

[17] Msadek N., Soua R., Engel T. Iot device fingerprinting: Machine learning based encrypted traffic analysis. Proceedings of the 2019 IEEE Wireless Communications and Networking Conference (WCNC).

[18] Sivanathan A., Sherratt D., Gharakheili H.H., Radford A., Wijenayake C., Vishwanath A., Sivaraman V. Characterizing and classifying IoT traffic in smart cities and campuses. Proceedings of the 2017 IEEE Conference on Computer Communications Workshops (INFOCOM WKSHPS).

[19] Marchal S., Miettinen M., Nguyen T.D., Sadeghi A.-R., Asokan N. (2019). Audi: Toward autonomous iot device-type identification using periodic communication. IEEE J. Sel. Areas Commun..

[20] Pinheiro A.J., de M. Bezerra J., Burgardt C.A.P., Campelo D.R. (2019). Identifying IoT devices and events based on packet length from encrypted traffic. Comput. Commun..

[21] Tekler Z.D., Low R., Zhou Y., Yuen C., Blessing L., Spanos C. (2020). Near-real-time plug load identification using low-frequency power data in office spaces: Experiments and applications. Appl. Energy.

[22] Duan C., Gao H., Song G., Yang J., Wang Z. (2021). ByteIoT: A Practical IoT Device Identification System based on Packet Length Distribution. IEEE Trans. Netw. Serv. Manag..

[23] Meidan Y., Bohadana M., Shabtai A., Guarnizo J.D., Ochoa M., Tippenhauer N.O., Elovici Y. ProfilIoT: A Machine Learning Approach for IoT Device Identification Based on Network Traffic Analysis. Proceedings of the Proceedings of the Symposium on Applied Computing.

[24] Yin F., Yang L., Wang Y., Dai J. IoT ETEI: End-to-End IoT Device Identification Method. Proceedings of the 2021 IEEE Conference on Dependable and Secure Computing (DSC).

[25] Perdisci R., Papastergiou T., Alrawi O., Antonakakis M. Iotfinder: Efficient large-scale identification of iot devices via passive dns traffic analysis. Proceedings of the 2020 IEEE European Symposium on Security and Privacy (EuroS&P).

[26] OConnor T., Mohamed R., Miettinen M., Enck W., Reaves B., Sadeghi A.-R. HomeSnitch: Behavior transparency and control for smart home IoT devices. Proceedings of the Proceedings of the 12th Conference on Security and Privacy in Wireless and Mobile Networks.

[27] Terrell J., Jeffay K., Smith F.D., Gogan J., Keller J. Passive, streaming inference of TCP connection structure for network server management. Proceedings of the International Workshop on Traffic Monitoring and Analysis.

[28] Alrawi O., Lever C., Antonakakis M., Monrose F. Sok: Security evaluation of home-based iot deployments. Proceedings of the 2019 IEEE Symposium on Security and Privacy (sp).

[29] Trimananda R., Varmarken J., Markopoulou A., Demsky B. Packet-level signatures for smart home devices. Proceedings of the Network and Distributed Systems Security (NDSS) Symposium.

[30] Hafeez I., Antikainen M., Ding A.Y., Tarkoma S. (2020). IoT-KEEPER: Detecting malicious IoT network activity using online traffic analysis at the edge. IEEE Trans. Netw. Serv. Manag..

[31] Ortiz J., Crawford C., Le F. DeviceMien: Network device behavior modeling for identifying unknown IoT devices. Proceedings of the Proceedings of the International Conference on Internet of Things Design and Implementation.

[32] LeCun Y., Bengio Y., Hinton G. (2015). Deep learning. Nature.

[33] Schmidhuber J. (2015). Deep learning in neural networks: An overview. Neural Netw..

[34] Rezaei S., Liu X. (2019). Deep Learning for Encrypted Traffic Classification: An Overview. IEEE Commun. Mag..

[35] Rezaei S., Liu X. (2018). How to achieve high classification accuracy with just a few labels: A semi-supervised approach using sampled packets. arXiv.

[36] Gunasekaran H., Ramalakshmi K., Arokiaraj A.R.M., Kanmani S., Venkatesan C., Dhas C.S.G. (2021). Analysis of DNA Sequence Classification Using CNN and Hybrid Models. Comput. Math. Methods Med..

[37] Li F., Liu M., Zhao Y., Kong L., Dong L., Liu X., Hui M. (2019). Feature extraction and classification of heart sound using 1D convolutional neural networks. EURASIP J. Adv. Signal Process..

